# Comparative Evaluation of Osseodensification Versus Conventional Osteotomy Technique on Dental Implant Primary Stability: An Ex Vivo Study

**DOI:** 10.7759/cureus.30843

**Published:** 2022-10-29

**Authors:** Vinod Bandela, Neema Shetty, Bharathi Munagapati, Ram B Basany, Saraswathi Kanaparthi

**Affiliations:** 1 Prosthodontics, Faculty of Dentistry, Pacific Academy of Higher Education and Research University, Rajasthan, IND; 2 Periodontics, Faculty of Dentistry, Pacific Academy of Higher Education and Research University, Rajasthan, IND; 3 Prosthodontics, GPR Dental College & Hospital, Kurnool, IND; 4 Prosthodontics, Mallareddy Dental College for Women, Hyderabad, IND; 5 Pedodontics and Preventive Dentistry, St. Joseph’s Dental College and Hospital, Eluru, IND

**Keywords:** resonance frequency analysis, primary stability, osseodensification, insertion torque, implant stability quotient, implant stability

## Abstract

Purpose

One of the commonest treatment options for replacing missing teeth is a root-form implant. Clinically, the key mechanical factor in achieving success is primary stability. This ex vivo study aims to evaluate whether osseodensification method will achieve good primary stability or the conventional drilling protocol.

Methods

Fresh iliac bone of the sheep was selected similar to D3 and D4 bone densities. A total of 22 osteotomy sites were prepared in the bone sample, of which 11 were prepared by osseodensification method (test group) and other 11 by conventional undersized drilling (control group). Primary stability was measured using insertion torque (IT), resonance frequency analysis (RFA), and reverse torque values (RTVs) by measuring implant stability quotient (ISQ). The recorded data were statistically analyzed using Statistical Package for the Social Sciences (SPSS) Version 22.0. The differences between groups were compared using the Mann-Whitney U test and independent t-test. The Pearson correlation coefficient test was performed to measure the linear relationship between two variables. The statistical significance level was established at p<0.05.

Results

When the correlation among IT, RTV, and ISQ was measured, a statistically significant correlation between IT and RTV (p=0.001) and between IT and ISQ (p=0.0001) was observed. A statistically significant (p=0.014) correlation between RTV and ISQ was also found.

Conclusion

Osteotomy prepared by osseodensification method showed higher IT, RTV, and ISQ values than the conventional undersized group.

## Introduction

One of the effective therapeutic choices for the rehabilitation of partial or total edentulism has been demonstrated to be endosseous dental implants. The osseointegration process, which provides stability and long-term survival, is what leads to success. Implant stability is one of the requirements for clinical success. The two types of implant stability are biological stability (secondary stability), which results from osseointegration, and mechanical stability (primary stability [PS]) between the implant and bone [1]. The density of bone, surgical technique, implant thread design, and geometry are the main elements in enhancing implant PS [2-4]. The mechanical interaction between the exterior surface of the implant and the walls of the osteotomy site provides PS. Insertion torque (IT), a measurement of the implant's rotational friction and a wholly mechanical factor regarded by the clinician as a reliable predictor of PS, is one approach to assess PS [5]. The implant PS and host bone density have a direct impact on the peak IT. When compared to implants implanted with low IT values, high IT might dramatically enhance the initial bone-to-implant contact (BIC) percentage [6].

To strengthen the initial stability of an implant placed in the osteotomy, many procedures have been introduced and put into operation in regions with poor bone density and insufficient bone volume. Alveolar ridge expansion and/or ridge splitting procedures, which call for a succession of osteotomes, chisels, or screw-type expanders to expand or divide the osteotomy site, are used to control edentulous ridges with insufficient breadth. Bone drilling surgery is the most frequent osteotomy preparation. The conventional drilling procedure (CDP) employs drills with positive rake angles that remove bone deposits from the prepared osteotomy site in a clockwise orientation. To overcome the potential limitations of CDP, osseodensification (OD) - a novel additive drilling protocol in the counter-clockwise direction for the osteotomy preparation - was introduced by Salah Huwais in 2013 [7,8].

The concept behind this technique is to compress the cancellous bone around the revolving drills, which largely improves the situations with low bone volume by physically increasing the interlocking between the bone and the implant surface. Additionally, it helps the edentulous area's osteoblast nucleation. The Densah® (Versah, Jackson, MI, USA) bur, a customized bur with a negative rake angle that enhances bone density while generating the least amount of heat, is used to perform osteotomies. It is distinguished by its minimal plastic deformation of soft bone caused by rolling and sliding contact at the osteotomy. It is a bone non-extraction method that promotes bone densification while offering benefits of speed and tactile drill control during osteotomy. Traditional drills remove the bone, and osteotomies cause microfractures in the trabeculae that need time to heal; this results in delayed secondary implant stability. On the other hand, Densah bur condensing by compaction autografting facilitates bone preservation. This procedure improves implant PS and enhances bone density around the implant. This process improves the implant's biomechanical stability. These burs may be rotated either clockwise in the non-cutting direction to densify bone or clockwise in the cut direction to cut bone [7].

The OD also has drawbacks. In areas of thick or corticated bone, such as the anterior mandibular region, this procedure must be utilized with caution. The temperature has been seen to rise when using the drills, which might lead to osteoblast necrosis. Consequently, extensive irrigation should be used before an osteotomy [9].

Due to viscoelastic deformation during the OD operation, the spring-back phenomena (bone compaction) is seen. The residual strain in the bone generates compressive pressures against the implant in this method to boost BIC and PS, which also stimulates osteogenic activity. Higher reverse torque, which is a sign of excellent PS, is caused by the reverse compression given to the implant surface. Utilizing the pilot drill first, followed by the narrowest Densah bur, make the osteotomy 1 mm deeper than the ultimate implant length. Later, the drill motor is switched to operate in the other direction (counterclockwise) at a speed of 800-1,500 rpm while being heavily watered. By doing this, the bur enters the osteotomy site in a direction that causes densification. The Densah bur is advanced into the osteotomy until it reaches the desired depth. Next, the bur is allowed to stay in the osteotomy, while the drill motor is set to the reverse densifying mode to densify and to autograft the bone into the osteotomy wall [7,10].

The amount of data on OD are still somewhat limited. Therefore, studies are needed to compare the PS of implants put in soft bone utilizing densifying burs and smaller CDP. The current study aimed to compare the PS and reverse torque values (RTVs) obtained by conventional undersized osteotomy preparation and OD osteotomy preparation using Densah burs in soft bone, as well as to assess the effectiveness of OD technique in low-density bone for achieving good PS.

## Materials and methods

**Figure 1:**
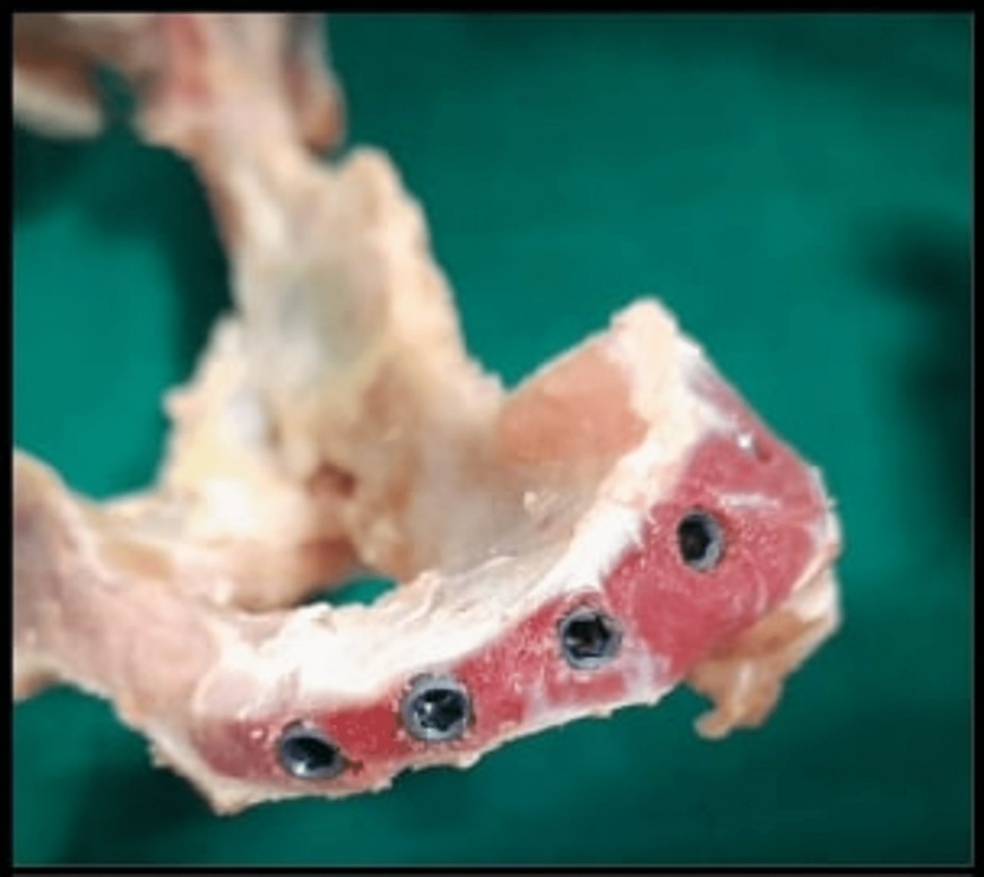
Bone model with implants

This ex vivo study was designed using fresh iliac bone of the sheep (Ovis aries) obtained from a local butcher similar to D3 and D4 bone densities that was tested by the tactile sensitivity of experienced implantologists. To expose the bone surface and prepare the bone samples, the soft tissue attachments were removed. On the iliac bone, 22 osteotomy sites were prepared, of which 11 were done by standard undersized drilling (control group) and 11 using the OD methodology (test group) (Figure 1).

All of the osteotomies in the control group were performed using the ADIN implant surgery kit (Alon Tavor, Galilee, Israel) and the manufacturer's suggested drilling sequence in order to receive dental implants measuring 4.2 mm x 10 mm (diameter x height) (Touareg™ S, Adin, Galilee, Israel). The last drill was not utilized in accordance with the soft bone undersized drilling technique. On the test group, all the 11 osteotomies were performed utilizing universal Densah surgical kit and standard Densah burs in accordance with the OD procedure for softer bone. Similar-sized implants were utilized. Drilling was done to a depth of 10mm using 2mm pilot drill with 1500rpm in a clockwise direction, followed by a second drill with Densah® bur VT1828 of 2.3mm in a counter-clockwise (CCW) direction at 1,500rpm and then the third drill with Densah bur VT2838 of 3.3mm in a CCW direction of similar rpm.

**Figure 2:**
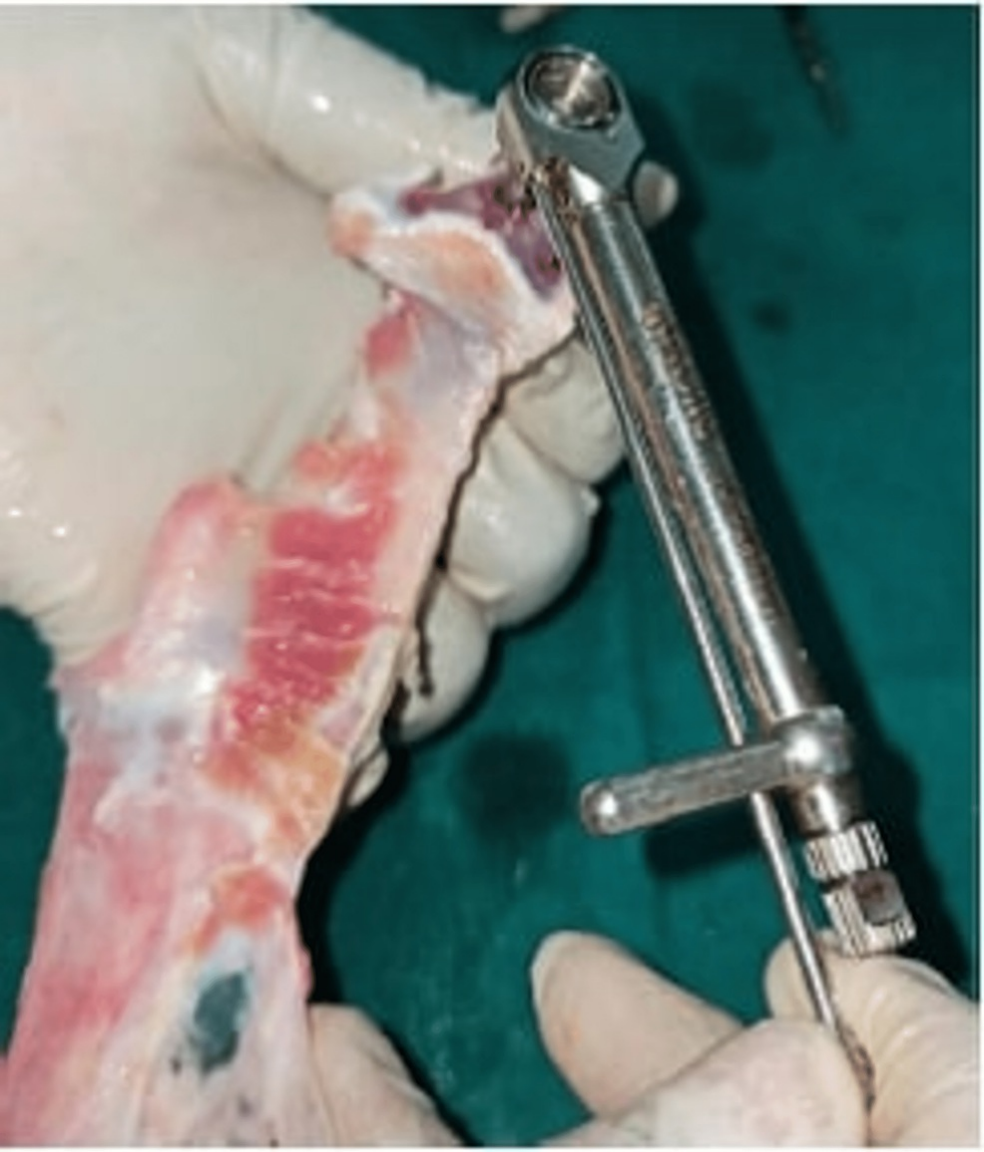
Recording insertion torque value with spring-style torque wrench

**Figure 3:**
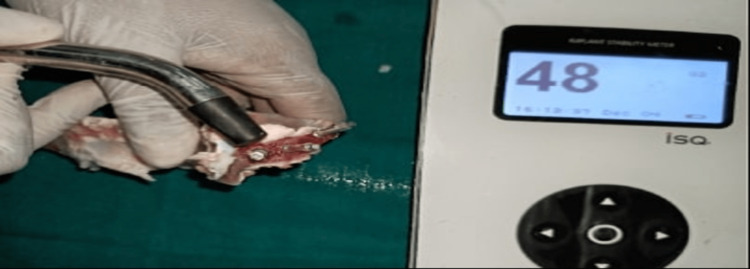
Recording resonance frequency analysis with the Osstell device

IT, resonance frequency analysis (RFA), and RTVs were used to assess PS. The peak value, measured in Newton centimeter (Ncm), was checked after the implant was placed using the manual ratchet (Figure 2). Osstell SmartPegs Type 49 specific to Adin implant system (Osstell AB, Gothenburg, Sweden) were placed onto the implant, and implant stability quotient (ISQ) values were recorded with the Osstell ISQ device (Osstell AB) to measure RFA (Figure 3). ISQ values were measured at four sites - mesial, distal, buccal, and lingual positions - and average values were recorded for each implant. Lastly, RTV were recorded by unwinding the implant in a CCW direction, and values were recorded manually by using the torque wrench/gauge for each implant placed. Values thus obtained were tabulated for both control and test groups. The data were recorded, and statistical analysis was carried out using Statistical Package for the Social Sciences (SPSS) Version 22.0 Software (IBM Corp., Armonk, NY, USA). The Mann-Whitney U test was used to compare the differences between two groups, independent t-test was used to compare the means between two groups, and Pearson correlation coefficient test was used to measure the strength of the linear relationship between two variables. The statistical significance level was established at p<0.05.

## Results

In this present study, comparison and evaluation of PS of implants placed by two different drilling methods were recorded using IT, ISQ, and RTVs.

When IT was compared between the control group and the test group, the mean IT for the control group was 34.0909 Ncm and that for the test group was 47.7273 Ncm, showing high IT by OD procedure. There was statistically significant difference (P=0.0004) between the test and control groups (Table 1).

**Table 1:** Comparison of insertion torque between the control group and the test group

Group	Mean	N	Std. Deviation	Median	Minimum	Maximum	Z-value	P-value
Control group	34.0909	11	2.02260	35.0000	30.00	35.00	-3.888	0.0004
Test group	47.7273	11	5.64076	50.0000	35.00	55.00		

When ISQ was compared between the control group and the test group, the mean ISQ value for the control group was 51.7727 and that for the test group was 62.8182. OD protocol showed higher ISQ values than the control group. There was statistically significant difference (P=0.0003) between the test and control groups (Table 2).

**Table 2:** Comparison of implant stability quotient between the control group and the test group

Group	Mean	N	Std. Deviation	Median	Minimum	Maximum	Z-value	P-value
Control group	51.7727	11	4.84440	51.0000	43.00	58.00	-6.453	0.0003
Test group	62.8182	11	2.96034	63.0000	58.00	67.00		

When RTVs were compared between the control group and test group, it was found that mean RTV was more in the test group (31.8182) than in the control group (18.6364). There was a statistically significant (p=0.0006) difference between the groups measured for reverse torque (Table 3).

**Table 3:** Comparison of reverse torque values between the control group and the test group

Group	Mean	N	Std. Deviation	Median	Minimum	Maximum	Z-value	P-value
Control group	18.6364	11	5.95437	20.0000	10.00	30.00	-4.443	0.0006
Test group	31.8182	11	7.83349	30.0000	15.00	45.00		

Table 4 shows the correlation between IT, RTV, and ISQ. There was a moderate positive correlation between IT and RTV, which was statistically significant (p=0.001), a high positive correlation between IT and ISQ, which was highly statistically significant (p=0.0001), and a moderate positive correlation between RTV and ISQ, which was statistically significant (p=0.014).

**Table 4:** Correlation among insertion torque, reverse torque value, and implant stability quotient

		Insertion Torque	Reverse Torque Value	Implant Stability Quotient
Insertion torque	Pearson correlation	1	0.641	0.706
	Sig. (two-tailed)	-	0.001	0.0001
Reverse torque value	Pearson correlation	0.641	1	0.514
	Sig. (two-tailed)	0.001	-	0.014
Implant stability quotient	Pearson correlation	0.706	0.514	1
	Sig. (two-tailed)	0.0001	0.014	-

## Discussion

In dentistry, implants have emerged as a viable procedure for replacing lost teeth. Constraints relating to the patient and those specific to the treatment are necessary for dental implant surgery to be effective. One of the most crucial elements in predicting implant success is proper treatment planning, which is based on the host characteristics, surgical considerations, and radiographic imaging modalities [11,12]. Direct BIC at the microscopic level and the quality and quantity of the histologic structure at the bone-implant interface, which significantly indicates better bone density, are two often mentioned variables impacting osseointegration. These are regarded as necessary conditions for implant loading and sustained clinical success. Therefore, it is crucial to maintain bone vitality and bone mass throughout the preparation of an osteotomy to ensure effective osseointegration [13,14].

In 1985, Lekholm and Zarb classified bone qualities into type I, type II, type III, and type IV. Based on microscopic cortical and trabecular bone features, Misch defined four categories of bone density as D1, D2, D3, and D4 in 1988 [11,15]. D3 and D4 bone types require longer healing time due to poor bone density and less BIC, but D1 and D2 bone types require less healing time and can be loaded right away. IT of more than 25 Ncm is thought to be necessary for a successful implant. However, in the event of an instantaneous loading, an IT of at least 32 Ncm is needed, with the possibility of an increase to 45 Ncm in regions with comparatively poor bone density. Higher PS that promotes contact osteogenesis may be easily attained in denser bone. However, PS is frequently challenging to attain in low-density bone. Early instability has several negative impacts, including distant osteogenesis, a protracted healing process, and a lower success rate. Due to insufficient bone volume surrounding the implant surface in such bone quality, it is challenging to achieve optimal PS for initial loading operations attaining higher than 35 N cm. This might lead to an early implant failure rate. In order to overcome the implant failure rate and to increase the success rate, modifications must be made in the implant design, drilling protocol, and implant bed preparation [16]. Undersized drilling, bicortical fixation, stepwise osteotomy, osteotome procedure, and OD utilizing densifying burs are a few methods to enhance the osteotomy site. Dr. Robert Summers' osteotome method aimed to compress the bone mechanically along the osteotomy walls with cylinder-shaped steel tools. As a result of the bone compaction caused by this procedure, the residual bone will be preserved as much as possible. Since the drill slices and removes the bone tissue from the osteotomy, conventional drilling is regarded as a subtractive operation. As bone is removed from the implantation bed, this has a significant impact on the main stability of the implant [5]. As a bone-conserving method, OD - a relatively recent approach to promote osteotomy site densification - has been presented. The use of the Densah burs in this operation permits bone preservation, bone condensation by compaction, and autografting during osteotomy preparation, increasing bone density in the peri-implant region and enhancing the mechanical stability of an implant. It takes more than 12 weeks to completely repair the bone at the osteotomy site using standard drilling [8,17]. In the current study, OD was performed, which helps in preserving the bone bulk and increases the density, thereby shortening the healing period. Similar to our study, Tian et al., compared the osseointegration of endosteal implants placed in atrophic mandibular alveolar ridges with alveolar ridge expansion surgical protocol. They showed OD drilling had better osseointegration than the control group [18]. Markovic et al. compared the primary and secondary stability of implants placed by bone condensing versus standard drilling and showed bone-condensing technique to be a good procedure to be followed to achieve greater implant stability in the posterior maxilla [19].

In the current study, we did conventional undersizing of the osteotomy site, since it is the most routinely used drilling protocol in preparing the osteotomy. Similarly, Alghamdi et al. evaluated the success rate of implants placed by using undersized implant site preparation in poor density bone [20].

In the present ex vivo study, the PS was investigated using sheep’s iliac crest model, which was in accordance with the other studies. When Oliveira et al. tested whether OD method affected IPS in sheep's iliac crest, they discovered that it rose by approximately 60% in comparison to standard drilling [21]. Lahens et al. looked into how OD affected the early osseointegration and initial stability of conical and parallel-walled endosteal implants in low-density bone using a sheep hip model [14]. The iliac crest bone of sheep, which primarily consists of porous trabecular bone with a thin cortical layer, was the subject of studies by Jimbo et al. and Galli et al., who examined the role of the undersized drilling technique in low-density bone. This model is therefore ideally suited to assess the bone healing responses in low-density bone [22,23].

In the present study, conventional undersized drilling and OD protocol was performed. Similar to our study, Slete et al. histomorphometrically compared osteotomy preparation through standard drilling, Summer’s osteotomes, and OD. The OD method produced 50% or more BIC percentage than with the other methods [24].

There are different methods to assess implant PS. They can be grouped as invasive/destructive methods and non-invasive/non-destructive methods. IT is a measure of the rotational friction of the implant, perceived by the clinician as a good estimator for PS. RFA is based on the resonance frequency of implant-bone complex at the time of implant placement. This method is expensive and technique sensitive. Therefore, in the current study, we measured the PS by IT, RFA, and RTV [11,25].

In the present study, OD group showed higher IT values compared with conventional undersized drilling group. The results were in agreement with other studies [8,26,27].

In the present study, OD showed higher ISQ values than the control group. The results were in accordance with the study by Degidi et al. They compared the effect of three drilling protocols of implant placement in bovine bone. The mean peak IT and VTW showed statistically significant improvement with the 10% undersized protocol, and increase in ISQ values [28].

In the present study, the OD group showed higher RTV than conventional undersized drilling group. The results were in agreement with those of Trisi et al., wherein the effect of OD on Cortex® implants installed over the sheep iliac crest showed high RT values for the OD protocol [10].

In the present study, there was a moderate positive correlation between IT and RT, which was statistically significant (p<0.05), a high positive correlation between IT and ISQ, which was highly significant (p<0.01), and a moderate positive correlation between RT and ISQ, which was statistically significant (p<0.01). Barberá-Millán et al. compared the PS of implants with OD vs underdrilling based on implant IT and RFA measurements and found IT and RFA values to be significantly higher in the OD group [29].

In the present study, IT and RFA showed a highly significant correlation. Similar to our study results, Turkyilmaz et al. showed a strong correlation between bone density, IT, and RFA values at implant placed in the posterior maxilla [30].

The present study also showed higher IT, RTV, and RFA values in the OD group than conventional drilling group. Cáceres et al. showed higher IT, RTV, and ISQ values on low-density bone by OD Densah protocol compared to standard drilling for BioHorizons® implants [31].

Limitations

The limitations of the present study include the small sample size. In addition, bone density evaluation with CBCT technique would have been a better measure, and IT and RTVs were recorded manually.

## Conclusions

To achieve good osseointegration in low-density bone, a newer technique of drilling, OD, would be of greater help. The results in the present study demonstrated higher IT, reverse torque, and ISQ values with OD protocol than conventional undersized protocol. Further investigations with increased sample size and more in vivo studies are needed to validate the clinical advantage of the OD technique, which, if proven, can serve as an efficient, accessible, and affordable technique that can fasten and improve the quality of osseointegration with endosseous dental implants even in compromised bone conditions.

## References

[1] Huang HM, Chee TJ, Lew WZ, Feng SW (2020). Modified surgical drilling protocols influence osseointegration performance and predict value of implant stability parameters during implant healing process. Clin Oral Investig.

[2] Albrektsson T, Zarb G, Worthington P, Eriksson AR (1986). The long-term efficacy of currently used dental implants: a review and proposed criteria of success. Int J Oral Maxillofac Implants.

[3] Bandela V, Munagapati B, Komala J, Basany RB, Patil SR, Kanaparthi S (2020). Comparison of primary stability of implants installed by two different methods in D3 and D4 bone types: an in vitro study. J Int Soc Prev Community Dent.

[4] Bergamo ET, Zahoui A, Barrera RB, Huwais S, Coelho PG, Karateew ED, Bonfante EA (2021). Osseodensification effect on implants primary and secondary stability: Multicenter controlled clinical trial. Clin Implant Dent Relat Res.

[5] Farronato D, Manfredini M, Stocchero M, Caccia M, Azzi L, Farronato M (2020). Influence of bone quality, drilling protocol, implant diameter/length on primary stability: an in vitro comparative study on insertion torque and resonance frequency analysis. J Oral Implantol.

[6] Podaropoulos L (2017). Increasing the stability of dental implants: the concept of osseodensification. Balk J Dent Med.

[7] Gaikwad AM, Joshi AA, Nadgere JB (2022). Biomechanical and histomorphometric analysis of endosteal implants placed by using the osseodensification technique in animal models: a systematic review and meta-analysis. J Prosthet Dent.

[8] Huwais S, Meyer EG (2017). A novel osseous densification approach in implant osteotomy preparation to increase biomechanical primary stability, bone mineral density, and bone-to-implant contact. Int J Oral Maxillofac Implants.

[9] Padhye NM, Padhye AM, Bhatavadekar NB (2020). Osseodensification -- a systematic review and qualitative analysis of published literature. J Oral Biol Craniofac Res.

[10] Trisi P, Berardini M, Falco A, Podaliri Vulpiani M (2016). New osseodensification implant site preparation method to increase bone density in low-density bone: In vivo evaluation in sheep. Implant Dent.

[11] Bandela V, Munagapati B, Komala J, Basany RB, Patil SR, Kanaparthi S (2020). Evaluating the primary stability of implants by two different insertion methods in compromised bone - a pilot study. Biomed Pharmacol J.

[12] Bajaj G, Bathiya A, Gade JK, Mahale Y, Ulemale M, Atulkar M (2017). Primary verses secondary implant stability in immediate and early loaded implants. Int J Oral Health Med Res.

[13] Tretto PH, Fabris V, Cericato GO, Sarkis-Onofre R, Bacchi A (2019). Does the instrument used for the implant site preparation influence the bone-implant interface? A systematic review of clinical and animal studies. Int J Oral Maxillofac Surg.

[14] Lahens B, Neiva R, Tovar N (2016). Biomechanical and histologic basis of osseodensification drilling for endosteal implant placement in low density bone. An experimental study in sheep. J Mech Behav Biomed Mater.

[15] Al-Sabbagh M, Eldomiaty W, Khabbaz Y (2019). Can osseointegration be achieved without primary stability?. Dent Clin North Am.

[16] Witek L, Neiva R, Alifarag A (2019). Absence of healing impairment in osteotomies prepared via osseodensification drilling. Int J Periodontics Restorative Dent.

[17] Koutouzis T, Huwais S, Hasan F, Trahan W, Waldrop T, Neiva R (2019). Alveolar ridge expansion by osseodensification-mediated plastic deformation and compaction autografting: a multicenter retrospective study. Implant Dent.

[18] Tian JH, Neiva R, Coelho PG (2019). Alveolar ridge expansion: comparison of osseodensification and conventional osteotome techniques. J Craniofac Surg.

[19] Marković A, Calasan D, Colić S, Stojčev-Stajčić L, Janjić B, Mišić T (2011). Implant stability in posterior maxilla: bone-condensing versus bone-drilling: a clinical study. Oral Surg Oral Med Oral Pathol Oral Radiol Endod.

[20] Alghamdi H, Anand PS, Anil S (2011). Undersized implant site preparation to enhance primary implant stability in poor bone density: a prospective clinical study. J Oral Maxillofac Surg.

[21] Oliveira PG, Bergamo ET, Neiva R, Bonfante EA, Witek L, Tovar N, Coelho PG (2018). Osseodensification outperforms conventional implant subtractive instrumentation: A study in sheep. Mater Sci Eng C Mater Biol Appl.

[22] Jimbo R, Giro G, Marin C (2013). Simplified drilling technique does not decrease dental implant osseointegration: a preliminary report. J Periodontol.

[23] Galli S, Jimbo R, Tovar N, Yoo DY, Anchieta RB, Yamaguchi S, Coelho PG (2015). The effect of osteotomy dimension on osseointegration to resorbable media-treated implants: a study in the sheep. J Biomater Appl.

[24] Slete FB, Olin P, Prasad H (2018). Histomorphometric comparison of 3 osteotomy techniques. Implant Dent.

[25] Swami V, Vijayaraghavan V, Swami V (2016). Current trends to measure implant stability. J Indian Prosthodont Soc.

[26] Delgado-Ruiz R, Gold J, Somohano Marquez T, Romanos G (2020). Under-drilling versus hybrid osseodensification technique: differences in implant primary stability and bone density of the implant bed walls. Materials (Basel).

[27] Lahens B, Lopez CD, Neiva RF (2019). The effect of osseodensification drilling for endosteal implants with different surface treatments: A study in sheep. J Biomed Mater Res B Appl Biomater.

[28] Degidi M, Daprile G, Piattelli A (2015). Influence of underpreparation on primary stability of implants inserted in poor quality bone sites: an in vitro study. J Oral Maxillofac Surg.

[29] Barberá-Millán J, Larrazábal-Morón C, Enciso-Ripoll JJ, Pérez-Pevida E, Chávarri-Prado D, Gómez-Adrián MD (2021). Evaluation of the primary stability in dental implants placed in low density bone with a new drilling technique, Osseodensification: an in vitro study. Med Oral Patol Oral Cir Bucal.

[30] Turkyilmaz I, Aksoy U, McGlumphy EA (2008). Two alternative surgical techniques for enhancing primary implant stability in the posterior maxilla: a clinical study including bone density, insertion torque, and resonance frequency analysis data. Clin Implant Dent Relat Res.

[31] Cáceres F, Troncoso C, Silva R, Pinto N (2020). Effects of osseodensification protocol on insertion, removal torques, and resonance frequency analysis of BioHorizons® conical implants. An ex vivo study. J Oral Biol Craniofac Res.

